# General knowledge of Jordanian healthcare providers about burn first aid-management

**DOI:** 10.1097/MD.0000000000045677

**Published:** 2025-10-31

**Authors:** Motaz Ghazi Qasaimeh, Ahlam J. Alhemedi, Dua Nassr Murrar, Ghayda’ Bani Irshaid, Malak Hassib Al-Attari, Ruba Hasan Al-Zaidan

**Affiliations:** a Department of General Surgery, Anesthesia and Urology, Faculty of Medicine, The Hashemite University, Zarqa, Jordan; b Department of Public Health and Family Medicine, Faculty of Medicine, Jordan University of Science and Technology, Irbid, Jordan.

**Keywords:** burn, first aid, healthcare providers, Jordan, knowledge

## Abstract

First aid is defined as emergency procedure that taken by individual to preserve the life of other individuals when they suddenly injured and it aims to reduce the complications of these injuries. The aim of this study was to assess the level of knowledge about burn first aid management among Jordanian healthcare providers. A cross-sectional study was carried out between February and July 2024 using a sample of healthcare professionals who are currently working in Jordan. Multiple logistic regression was performed to define predictors of good knowledge of burn. A total of 383 participants were included in the analysis. The total mean score for the sample was 26.58 ± 4.05 out of 33, indicating a moderate level of knowledge. A total of 52.1% had a poor knowledge. Gender shows a borderline significant association, with females having lower odds compared with males (odds ratios [OR] = 0.64, 95% confidence intervals [CI]: 0.41–1.00, *P* = .05). Additionally, specialty is a significant predictor: general practitioners (OR = 3.24, 95% CI: 1.24–8.49, *P* = .017), family medicine specialists (OR = 3.76, 95% CI: 1.37–10.36, *P* = .010), and general surgeons (OR = 8.96, 95% CI: 1.85–43.33, *P* = .006) have significantly higher knowledge compared with nonphysician. This study showed a moderate general understanding of burn first aid among healthcare providers, with more than half exhibiting inadequate knowledge. Gender and specialty were recognized as significant determinants, with males and physicians, especially general surgeons, demonstrating elevated levels of expertise.

## 1. Introduction

First aid is defined as emergency procedure that taken by individual to preserve the life of other individuals when they suddenly injured and it aims to reduce the complications of these injuries. During the process of training in providing first aid, several actions must be taken into account, including full knowledge of the importance of these procedures in providing the best care for the injured person and requesting help when needed when these first aids are not sufficient to preserve the life of the injured person. On the other hand, the provider of these services must ensure that the tools used are ready and comply with medical standards. Also, awareness of first aid must be increased so that any person from the society can provide it when needed.^[1]^ One of the injuries that require first aid is burns. Burns are a worldwide public health issue that accounts for ~180,000 deaths each year. Middle- and low-income countries such as Jordan account for the majority of these events.^[2]^ The risk of burns is higher for individuals residing in low- and middle-income countries than for those residing in high-income countries with an estimated mean total healthcare cost per burn patient of 88,218 United State dollars. Burn risk is, however, correlated with socioeconomic status in all countries.^[2]^ Previous research in Jordan found that the mean duration of hospital stays for burn patients ranged between 16 and 18 days and the estimated mortality rate was 8.2% to 14.6%.^[3,4]^

Burns are considered one of the most common injuries in emergency rooms, as they require immediate evaluation and treatment from service providers. Burns range from superficial burns to life-threatening burns and they occur when body skin exposed to high temperatures, friction, radiation and chemicals, or electrical sources.^[5,6]^ Burns cause several complications, including loss of a large amount of body fluids, ease of infection, tissue damage, and other problems.^[7,8]^ When providing the necessary first aids to a person who exposed to burns, there are some steps that must be followed to preserve the person’s life and reduce injuries to his body. These procedures include removing clothing from the injured area and providing the affected area with water. If the burn covers a large part of the skin area, then the service provider can use a blanket or fire extinguisher. When person exposed to burns resulting from chemicals, the solution is to dilute these materials by increasing the amount of water. The most important thing is that these steps reduce the severity of the burn, and the first aid provider must immediately transfer the person to the nearest health center or hospital.^[9]^ Therefore, there is an urgent need to increase the awareness of how to deal with such emergency cases to avoid many of the complications that result from them. This, in turn, enhances the role of caregivers and those responsible for providing first aid, as they provide initial care to preserve the life of the injured person and reduce complications resulting from burn injuries by providing rapid treatment in a timely manner. The aim of this study was to assess the level of knowledge about burn first aid management among Jordanian healthcare providers.

## 2. Methods

### 2.1. Study design and population

A cross-sectional study was carried out between February and July 2024 using a sample of healthcare professionals who are currently working in Jordan.

### 2.2. Study settings and patients’ recruitment

Study participants were healthcare professionals who are currently practicing their profession in Jordan. Healthcare professionals were invited to participate in this study using convenience sampling technique. Healthcare professionals were approached through social media platforms (WhatsApp and Facebook). The survey link was distributed through WhatsApp and Facebook groups associated with King Abdullah Hospital, Basmah Hospital, nurses, emergency physicians, and family medicine physicians affiliated to Jordan University of Jordan, Hashemite University, physicians affiliated to Ministry of Health.

The inclusion criteria for eligibility are as follows: healthcare professionals (medical doctors, nurses, and paramedics), who are currently practicing their profession in Jordan. Exclusion criteria were other healthcare professionals (ophthalmologist, radiologist, psychiatrists, neurologist, and forensic medicine specialists) or those who are not currently practicing in Jordan.

### 2.3. Data collection instrument

Data were collected using a well-designed questionnaire composed of different sections based on extensive literature review.^[10–14]^ These sections were chosen and organized based on a review of similar literature. Healthcare professionals completed the self-administered anonymous questionnaire in the Arabic language.

The questionnaire instrument for this study consisted of 6 sections. The first section included demographic and practice characteristics questions (gender, age, years of experience, number of patients seen, place of encountering patients, occupation, specialty, and level of occupation). The second section included questions on knowledge about burn degrees and causes (6 questions). The third section included questions on knowledge items related to burn treatment (10 questions). The fourth section included questions on knowledge items related to the burn injury care (four questions). The fifth section included questions on criteria for referral of patients to a burn unit (eight questions). The sixth section included questions on factors determine the morbidity and mortality in patients with burn injuries (5 questions). The total number of questions used to assess participants burn knowledge was 33 questions (yes/no format). For each correct answer, the participants were given 1 point with a maximum of 33. The higher the score, the higher the burn knowledge.

### 2.4. Questionnaire validity and piloting phase

Two clinicians from the Jordan University of Science and Technology and Hashemite University evaluated and validated the questionnaire instrument. They were questioned about the clarity and comprehensibility of the questionnaire items, as well as their face validity and whether any were difficult to understand. Subsequently, a pilot study was conducted on 30 participants who satisfied the study’s inclusion criteria. The participants were questioned regarding the comprehensibility and clarity of the questionnaire, as well as whether any of the inquiries were difficult to comprehend. They verified that the questionnaire was deemed straightforward to comprehend and complete. The overall Cronbach alpha measure was 0.77, which is identified as acceptable stability for the questionnaire instrument.^[15]^

### 2.5. Ethical approval

This study was approved by the Institutional Review Board at The Hashemite University (Ref: 13/3/2023/2024). All healthcare professionals provided their consent before participating in this study.

### 2.6. Sample size

The target sample size was estimated using a population size of 35,000 physicians in Jordan.^[16]^ With a confidence interval (CI) of 95%, a standard deviation of 0.5, and a margin of error of 5%, the minimum required sample size was 379 participants.

### 2.7. Data analysis

Data analyses were performed using the Statistical Package of Social Sciences software Version 29.0). Descriptive statistics such as the frequency and percentage were used for categorical variables. The mean and the standard deviation were used to present the continuous variables. The mean burn knowledge score (26.58 ± 4.05) was used as the cutoff point to define poor and good knowledge. Therefore, any participant scored 26.58 and above was categorized as having good burn knowledge and those who score below 26.58 was categorized as having poor burn knowledge. Multiple logistic regression was performed to define predictors of good knowledge of burn. Participants’ sociodemographic characteristics served as the independent variables in the regression model. The mean burn knowledge score served as the dependent variable in the regression model. The findings of the regression analysis were presented as odds ratios (OR) with 95% CIs. The level of significance was defined as α = 0.05.

## 3. Results

A total of 383 participants were included in the analysis. Most of participants (n = 215, 56.1%) were females, while 168 participants (43.9%) were male. The majority of the participants (n = 259, 67.6%) were aged <30 years old, followed by 89 participants (23.2%) aged 30 to 40 year. In term of occupation, a total of 292 participants were a medical doctor (76.2%), followed by nurse (n = 83, 21.7%). The majority of the participants (n = 216, 56.4%) had an experience <2 years, and only 63 (16.4%) participants had more than 10 years of experience. Most of participants (n = 191, 49.9%) had <10 patients per shift, while 25 participates (6.5%) had more than 51 patients per shift. Additional details about participants’ demographics are provided in Table 1.

**Table 1:** The demographic characteristics of the participants (n = 383).

Demographic variable		Frequency	Percentage
Gender	Male	168	43.9%
	Female	215	56.1%
Age	<30 yr	259	67.6%
	30–40 yr	89	23.2%
	>40 yr	35	9.1%
Years of experience	<2 yr	216	56.4%
	2–5 yr	70	18.3%
	6–10 yr	34	8.9%
	>10 yr	63	16.4%
Number of patients seen	Zero	95	24.8%
	<10	191	49.9%
	10–50	72	18.8%
	51–100	16	4.2%
	>100	9	2.3%
Place of encountering patients	As outpatient in the clinic	69	18.0%
	As outpatient in emergency room	114	29.8%
	As inpatient in the hospital	74	19.3%
	As outpatient in the clinic and emergency room	41	10.7%
	As inpatient admission in the hospital, and as outpatient in the clinic and emergency room	30	7.8%
	As inpatient admission and as outpatient in clinic	13	3.4%
	As inpatient admission in the hospital and as outpatient in the emergency room	42	11.0%
Occupation	Medical doctor	292	76.2%
	Nurse	83	21.7%
	Paramedic	8	2.1%
Specialty	I am not a physician	91	23.8%
	General practitioner	157	41.0%
	Family medicine	75	19.6%
	Emergency medicine	3	0.8%
	Internal medicine	9	2.3%
	General surgery	17	4.4%
	Other specialties	31	8.1%
Level of occupation	I am not a physician	91	23.8%
	General practitioner	64	16.7%
	Intern doctor	96	25.1%
	Resident	92	24.0%
	Specialist	40	10.4%

The knowledge of burn was assessed using 5 items, the first item was related to the burn degrees and causes. The majority of the participants (n = 365, 95.05%) agreed that first degree burn is painful, while 289 participants (75.26%) reported that it is whitish in color. Most participants (n = 344, 89.58%) agreed that the most common cause for burns in children is hot liquids, while only 145 (37.76%) participants reported that the most common cause of burn in adult is chemical burn Table 2.

**Table 2:** Knowledge items related to burn degrees and causes (n = 383).

According to your knowledge about burn degrees and causes, please answer the following statements	No	Yes
First degree burn is painful	18 (4.95%)	365 (95.05%)
First degree burn is whitish in color	94 (24.74%)	289 (75.26%)
Second degree burn usually involves blisters	18 (4.95%)	365 (95.05%)
The most common cause for burns in children is hot liquids	39 (10.42%)	344 (89.58%)
The most common cause of burn in adult is chemical burn	238 (62.24%)	145 (37.76%)
Symptoms of compartment syndrome include pain, paranesthesia, pallor, no pulse, paralysis	56 (14.84%)	327 (85.16%)

Regarding the burn treatment, the majority of the participants (n = 349, 90.89%) agreed that removing of jeweler and clothes should be immediately. Notably, a total of 351 participants (91.41%) reported that the rule of nine, palm size, and other charts can be used to calculate total body surface area (TBSA) involved. Regarding the fluids to be given for burn patient, 202 participants (52.60%) reported that should be a normal saline, while 181 participants (47.40%) disagreed (Table 3).

**Table 3:** Knowledge items related to burn treatment (n = 383).

For burn treatment, answer the following questions	No	Yes
You should do Immediate removal of jeweler and clothes	34 (9.11%)	349 (90.89%)
The rule of nine, palm size, and other charts can be used to calculate total body surface area involved	32 (8.59%)	351 (91.41%)
Each body part has the same percentage of surface area in both adults and children	56 (14.84%)	327 (85.16%)
Best type of fluids to be given for burn patient is Normal Saline	181 (47.40%)	202 (52.60%)
There are special formulas to calculate fluids requirements in burn injuries	23 (6.25%)	360 (93.75%)
All patients with a suspicion of inhalation injury should be intubated	154 (40.36%)	229 (59.64%)
Systemic antibiotics for all burn injuries is recommended	156 (40.89%)	227 (59.11%)
Tetanus vaccine should be considered.	130 (34.11%)	253 (65.89%)
For patients with severe burn urine output should be monitored	18 (4.95%)	365 (95.05%)
First aid irrigation for the burned wound should be at least 20 min	105 (27.60%)	278 (72.40%)

Regarding the burn injury care, cleaning the burn area initially with aseptic solutions was reported by 83 (21.61%) participants, while dressing using topical antimicrobial usually used for burned area was agreed by 324 participants (84.38%). Furthermore, 344 participants agreed that surgical debridement sometimes is needed. Additional details about the burn injury care are provided in Table 4.

**Table 4:** Knowledge items related to the burn injury care (n = 383).

For burn injury care, please answer the following	No	Yes
Cleaning the burn area initially with aseptic solutions	300 (78.39%)	83 (21.61%)
Dressing using topical antimicrobial usually used for burned area	59 (15.63%)	324 (84.38%)
Surgical debridement sometimes is needed	39 (10.42%)	344 (89.58%)
One of the common topical antimicrobials used in burn is silver sulfadiazine	67 (17.71%)	316 (82.29%)

A total of 295 participants (76.82%) agreed that patients younger than 10 years and older than 50 years with burn of more than 10% of body surface area should be referred to a burn unit. However, burns to face, hands, feet, genitalia, and major joints are one of the criteria for referral of patients to a burn unit reported by 358 (93.23%) participants. Furthermore, suspicion of inhalational burn and circumferential deep burn to extremity or chest were reported by n = 340 (88.54%) and n = 279 (72.66%), respectively. Additional details about criteria for referral of patients to a burn unit are provided in Table 5.

**Table 5:** Criteria for referral of patients to a burn unit (n = 383).

Please choose the correct answer from the sentences below. The main criteria for referral of patients to a burn unit include:	No	Yes
Patients younger than 10 yr and older than 50 yr with burn of more than 10% of body surface area	88 (23.18%)	295 (76.82%)
Burns to face, hands, feet, genitalia, and major joints	25 (6.77%)	358 (93.23%)
Electrical burns	50 (13.28%)	333 (86.72%)
Suspicion of inhalational burn	45 (11.98%)	338 (88.02%)
Circumferential deep burn to extremity or chest	43 (11.46%)	340 (88.54%)
Chemical burns	104 (27.34%)	279 (72.66%)
Burn with trauma and high risk of mortality	37 (9.90%)	346 (90.10%)
All patients should be admitted	80 (21.09%)	303 (78.91%)

The majority of participants (n = 372, 96.88%) reported that TBSA of burn is a factor determining the morbidity and mortality in patients with burn injuries. Age of the patients and degree of burn were reported by n = 335 (87.24%) and n = 368 (95.83%) participants as factors that determine the morbidity and mortality in patients with burn injuries. Additional details about the factors that determine the morbidity and mortality in patients with burn injuries are provided in Table 6.

**Table 6:** The factors determine the morbidity and mortality in patients with burn injuries (n = 383).

Which of the following factors determine the morbidity and mortality in patients with burn injuries?	No	Yes
Total body surface area of burn	11 (3.13%)	372 (96.88%)
Age of the patient	48 (12.76%)	335 (87.24%)
Degree of burn	15 (4.17%)	368 (95.83%)
Associated trauma	39 (10.42%)	344 (89.58%)
Prescence of inhalational burn	28 (7.55%)	355 (92.45%)

The total mean score for the sample was 26.58 ± 4.05 out of 33, indicating a moderate level of knowledge. A total of 200 participants (52.1%) had a poor knowledge, and 184 participants had a good knowledge. Gender shows a borderline significant association, with females having lower odds compared with males (OR = 0.64, 95% CI: 0.41–1.00, *P* = .05). Additionally, specialty is a significant predictor: general practitioners (OR = 3.24, 95% CI: 1.24–8.49, *P* = .017), family medicine specialists (OR = 3.76, 95% CI: 1.37–10.36, *P* = .010), and general surgeons (OR = 8.96, 95% CI: 1.85–43.33, *P* = .006) have significantly higher knowledge compared with nonphysician. Additional details about factors that influence the knowledge level are provided in Table 7.

**Table 7:** Factors associated with knowledge score: multiple logistic regression.

Variables		OR (95% CI)	*P*-value
Gender	Male	Reference	
	Female	0.64 (0.41–1.00)	.051
Age	<30 yr	0.00 (0.00–0.00)	.411
	30–40 yr	1.65 (0.61–4.44)	.325
	>40 yr	0.96 (0.22–4.21)	.962
Years of experience	<2 yr	Reference	
	2–5 yr	1.05 (0.48–2.26)	.906
	6–10 yr	0.65 (0.18–2.33)	.511
	>10 yr	0.44 (0.10–1.89)	.269
Number of patients seen	Zero	Reference	
	<10	0.86 (0.50-1.50)	.604
	10–50	1.19 (0.58–2.44)	.645
	51–100	0.59 (0.14–2.38)	.455
	>100	0.20 (0.03–1.27)	.088
Place of encountering patients	As outpatient in the clinic	Reference	
	As outpatient in emergency room	1.10 (0.54–2.23)	.791
	As inpatient in the hospital	0.79 (0.36–1.75)	.566
	As outpatient in the clinic and emergency room	0.81 (0.35–1.90)	.633
	As inpatient admission in the hospital, and as outpatient in the clinic and emergency room	1.33 (0.47–3.72)	.589
	As inpatient admission and as outpatient in clinic	2.38 (0.61–9.24)	.209
	As inpatient admission in the hospital and as outpatient in the emergency room	1.28 (0.52–3.16)	.593
Occupation	Medical doctor	Reference	
	Nurse	1.42 (0.47–4.24)	.534
	Paramedic	2.31 (0.42–12.60)	.333
Specialty	I am not a physician	Reference	
	General practitioner	3.24 (1.24–8.49)	.017
	Family medicine	3.76 (1.37–10.36)	.010
	Emergency medicine	0.97 (0.07–12.90)	.981
	Internal medicine	0.66 (0.11–4.09)	.654
	General surgery	8.96 (1.85–43.33)	.006

## 4. Discussion

Burns are one of the most common problems that require first aid, as they cause many deaths in addition to the long-term complications that accompany them, which may sometimes lead to disability and deformities. Therefore, in this study, the aim was to measure the level of knowledge related to first aid and procedures for treating such an emergency case among healthcare providers.

In our study, the majority of the participants (95.05%) agreed that first degree burn is painful, while 75.26% reported that it is whitish in color. As known for the first degree of burn or as it called superficial burns make the skin color appear red, cause inflammation and swelling on the affected area, and can heal in ~1 week.^[17]^ First degree burn characterized by pain and irritation to the burned area because it affects only the epidermis which is contains sensory nerves endings.^[18,19]^ This result is not surprising and was logical compared with previous studies. In a study conducted in the Kingdom of Saudi Arabia to find out whether medical staff members were familiar with first aid, it was found that despite their knowledge of the importance of these procedures, awareness of them was very low, as first aid workshops on this subject were limited. In addition, most medical staff members resorted to traditional methods of treatment.^[20]^ In another study that was conducted in 2024 in Iraq, in which nurses participated, it became clear that they had very little to moderate knowledge of how to deal with a person who had been burned.^[21]^ This problem was not only present in the Middle East as in Brazil, for example, there was little awareness and education among physicians and nurses about providing first aids when a person with burns came to them.^[22]^ Thus, these studies confirm the lack of interest in this aspect of first aid, which is consistent with this result.

In our study, most participants (89.58%) agreed that the most common cause for burns in children is hot liquids, while only 37.76% participants reported that the most common cause of burn in adult is chemical burn. As for the main cause of burns among children, it became clear from the results of a study in Saudi Arabia that hot liquids were among the causes that led to burns in them. This study showed that the percentage of burns in this group is high compared with the elderly.^[23]^ As for the elderly, studies in Iran and China have revealed that the primary cause of burns among them was exposure to chemicals. This was due to the widespread presence of factories in advanced societies, where people were often exposed to these burns as a result of their work or when trying to handle chemicals incorrectly and this is consistent with the results we obtained in our study.^[24,25]^

In our study, regarding the burn treatment, the majority of the participants (90.89%) agreed that removing of jeweler and clothes should be immediately. Since the limited knowledge about first aid of burns among healthcare professionals, Fathuldeen et al and Kattan et al verified that more than half of them had knowledge of the procedure for removing clothing and jewelry when a person exposed to burns.^[10,11]^ In addition to that, our study found a total of 91.41% of the participants reported that the rule of nine, palm size, and other charts can be used to calculate TBSA involved. Regarding the fluids to be given for burn patient, 52.60% reported that should be a normal saline, while 47.40% disagreed. There are many methods that used by healthcare professionals to evaluate and calculate the surface of the affected area by burns. The most accurate one among them is Lund and Browder chart which use a chart that represents the front and back of human body.^[26]^ “Rule of Nine” is also used to calculate the overall area of body that affected by burns. The importance of this rule lies in calculating the amount of fluids lost to replace them when the patient is exposed to a burn.^[27]^ Another way is to use palm of the person who was exposed to the burn, not the person providing the first aid. This is done by making the parts of the hand close to each other, as the person’s hand represents 1% of the total area of the body.^[28]^ Another thing that found to be aligned with our study is among the fluids used in the process of fluid replacement, crystalloids such as Lactate Ringers and normal saline were the best choice for the primary treatment.^[29]^ Moreover, the World Health Organization placed Lactate Ringers and normal saline on the list of the best fluids to be given in emergency situations.^[30]^ In contrary, Greenhalgh disagreed with that result, as he found that normal saline was one of the least used fluids that used to replace the loss by burns.^[31]^

In our study, regarding the burn injury care, cleaning the burn area initially with aseptic solutions was reported by 21.61% of the participants, while dressing using topical antimicrobial usually used for burned area was agreed by 324 participants (84.38%). Furthermore, 344 participants agreed that surgical debridement sometimes is needed. This was reliable with a questionnaire distributed on healthcare professionals in Sri Lanka to measure their knowledge of the special cleaning process for burn wounds and how to deal with them; they were asked about antiseptic solutions and it was found that a large number of them preferred to use antiseptic materials such as alcohol to clean burn wounds.^[32]^ As person with burns is more susceptible to infection and therefore requires the use of certain antimicrobials. Results from a study conducted at some specialized burn treatment centers in the United States of America showed that the majority of service providers use topical antimicrobials routinely to prevent wound infection.^[33]^ On the hand, and related to surgical debridement, World Health Organization encourages surgical cleaning to save lives of people and reduce the complications of burns. However, most medical personnel lack these procedures, so the responsible authorities must ensure that their specialists have full knowledge of them.^[34]^ Another study conducted in the United Kingdom on some surgical operations showed that surgeons had preferable opinion on the importance of removing it and cleaning the wounds resulting from burns in order to remove the following tissues and prevent them from spreading to other parts of the body.^[35]^

In our study, a total of 295 participants (76.82%) agreed that patients younger than 10 years and older than 50 years with burn of more than 10% of body surface area should be referred to a burn unit. However, burns to face, hands, feet, genitalia, and major joints are one of the criteria for referral of patients to a burn unit reported by 93.23% of the participants. Back to the American Burn Association, this association has set some criteria by which the transfer of the burned person to the emergency unit is determined. One of these criteria was that if the patient is less than 10 years old and more than 50 years old and suffers from burns that cover more than 10% of the surface area of the body, in addition to burns that occur on the face, genitals, hands, feet, major joints, and perineum, then these burns are considered second- and third-degree injuries that require immediate admission to a health center or a specialized burn center.^[36]^ Furthermore, suspicion of inhalational burn and circumferential deep burn to extremity or chest were reported by 88.54% and 72.66%, respectively. There were 2 studies that confirm the conclusion drawn from our study. A study in Egypt to determine the prevalence of burns due to inhalation injuries, it was found that the rate of injuries and the prevalence of them are high compared with other injuries that can be caused by burns and also caused a high percentage of deaths. In another study, it was found that burns that occur in the extremities and on the chest are associated with abdominal compartment syndrome, which results in high rates of deaths due to the impact on the respiratory tract. It was proven that the number of patients who died due to it reached more than half.^[37,38]^

In our study, the majority of participants (96.88%) reported that TBSA of burn is a factor that determines the morbidity and mortality in patients with burn injuries. Age of the patients and degree of burn were reported by 87.24% and 95.83% of the participants as factors determine the morbidity and mortality in patients with burn injuries. Related to TBSA, a study conducted in Pakistan to determine the relationship between the it and the incidence of death, it was found that among people with burns, most of whom in this study had been involved in traffic accidents, and the increase in the TBSA of the body was directly related to the increase in the mortality rate, because these burns caused some complications such as severe infection, insufficient blood flow, and heart failure.^[39]^ Another study on children from South America, it was found that the children in this study had a percentage of the area exposed to the burns over 60% of the TBSA, which led to some complications that led to increased death rates within this group.^[40]^ According to age and to identify the relationship between it and mortality rates caused by burns, there was a study that showed that the 2 factors are linked to each other, as the elderly have higher mortality rates when exposed to severe injuries, especially second- and third-degree injuries.^[41]^ Studying the relationship between the degree of burn and the mortality rate, it was found that there is a relationship between the degree of burn and the area covered by the burn, such that the larger the area, the greater the degree of burn, and thus, the number of deaths increases in a proportional relationship, as which is what came in proportion to our result.^[42]^

In our study, the total mean knowledge score for the sample was 26.58 ± 4.05 out of 33, indicating a moderate level of knowledge. A total of 52.1% had a poor knowledge. This result was consistent with Studies examined in different places such as Saudi Arabia, India, and Ethiopia which showed that medical care providers need more workshops and education, as it was found that their knowledge of first aids related to burns is too little.^[20,43,44]^ Gender in our study shows a borderline significant association, with females having lower odds compared with males (OR = 0.64, 95% CI: 0.41–1.00, *P* = .05). When comparing the genders of health workers regarding knowledge of primary first aid of burns, it was found that males had higher knowledge than females.^[45]^ Additionally, specialty is a significant predictor: general practitioners (OR = 3.24, 95% CI: 1.24–8.49, *P* = .017), family medicine specialists (OR = 3.76, 95% CI: 1.37–10.36, *P* = .010), and general surgeons (OR = 8.96, 95% CI: 1.85–43.33, *P* = .006) have significantly higher knowledge compared with nonphysician. There was limited data related to this result, but there was a study in Saudi Arabia showed that physicians who deal with burns and provide first aid to those injured have more knowledge than those who do not deal with such cases. This was the first study in the Middle East to study the level of knowledge among physicians regarding to burn first aid.^[46]^

This study has limitations. The online cross-sectional survey study design has restricted ability to examine causality across the study variables. Besides, the generalizability of the study findings might have been affected as the study population is restricted to users of social media platforms. The small sample size for specific subgroups might have affected the statistical power for the study estimate. Moreover, self-administered study design is prone to reporting bias. The exclusion of healthcare providers’ attitudes and practices regarding burn first aid management is another limitation of this study. Despite that knowledge was evaluated, attitudes and practices are essential elements that significantly impact real-world applications. Therefore, the study findings should be interpreted carefully.

## 5. Conclusion

This study showed a moderate general understanding of burn first aid among healthcare providers, with more than half exhibiting inadequate knowledge. Gender and specialty were recognized as significant determinants, with males and physicians, especially general surgeons, demonstrating elevated levels of expertise. These findings underscore the pressing necessity for focused educational interventions and training initiatives. Increasing awareness via seminars may enhance preparedness and responsiveness in burn-related emergencies.

## Acknowledgments

We would like to thank The Hashemite University for their support.

## Author contributions

**Conceptualization:** Motaz Ghazi Qasaimeh.

**Data curation:** Motaz Ghazi Qasaimeh, Ahlam J. Alhemedi.

**Formal analysis:** Motaz Ghazi Qasaimeh.

**Funding acquisition:** Motaz Ghazi Qasaimeh.

**Investigation:** Motaz Ghazi Qasaimeh, Ahlam J. Alhemedi, Dua Nassr Murrar, Ghayda’ Bani Irshaid, Malak Hassib Al-Attari, Ruba Hasan Al-Zaidan.

**Methodology:** Motaz Ghazi Qasaimeh.

**Project administration:** Motaz Ghazi Qasaimeh, Ahlam J. Alhemedi.

**Resources:** Motaz Ghazi Qasaimeh, Ahlam J. Alhemedi, Dua Nassr Murrar, Ghayda’ Bani Irshaid, Malak Hassib Al-Attari, Ruba Hasan Al-Zaidan.

**Software:** Motaz Ghazi Qasaimeh.

**Supervision:** Motaz Ghazi Qasaimeh, Ahlam J. Alhemedi.

**Validation:** Motaz Ghazi Qasaimeh.

**Visualization:** Motaz Ghazi Qasaimeh.

**Writing – original draft:** Motaz Ghazi Qasaimeh, Ahlam J. Alhemedi.

**Writing – review & editing:** Motaz Ghazi Qasaimeh, Ahlam J. Alhemedi, Dua Nassr Murrar, Ghayda’ Bani Irshaid, Malak Hassib Al-Attari, Ruba Hasan Al-Zaidan.

## References

[1] Zideman DA Singletary EM Borra V. European resuscitation council guidelines 2021: first aid. Resuscitation. 2021;161:270–90. 33773828 10.1016/j.resuscitation.2021.02.013

[2] World Health Organization. Burns. 2023. https://www.who.int/news-room/fact-sheets/detail/burns. Accessed June 30, 2025.

[3] Bataineh ZA Al Quran TM Al Balas H Khammash MR. Pattern of burn injury at north of Jordan. Int J Burns Trauma. 2018;8:1–5. 29531853 PMC5840313

[4] El-Maaytah K. Patterns and Sequelae of burn injury at the Jordanian Royal Medical Services rehabilitation center in 2005-2017: a cross-sectional study. Electron Phys. 2019;11:7552–7.

[5] Ja G-E. Burns: definition, classification, pathophysiology and initial approach. Int J Gen Med. 2020;5:2327–5146.

[6] Vivó C Galeiras R del Caz MD. Initial evaluation and management of the critical burn patient. Med Intensiva. 2016;40:49–59. 26724246 10.1016/j.medin.2015.11.010

[7] Branski LK Herndon DN Barrow RE. 1 - A brief history of acute burn care management. In: Herndon DN, ed. Total Burn Care (Fifth Edition). Elsevier; 2018:1–7.e2.

[8] Lee KC Joory K Moiemen NS. History of burns: the past, present and the future. Burns & Trauma. 2014;2:169–80. 27574647 10.4103/2321-3868.143620 PMC4978094

[9] World Health Organization. Burns. 2023. https://www.who.int/news-room/fact-sheets/detail/burns. Accessed May 15, 2025.

[10] Fathuldeen AA Alduheim MA Alqahtani AS. Knowledge and practice of burn management among physicians using burn manikin in Ha’il, Kingdom of Saudi Arabia. Cureus. 2023;15:e36196. 37065347 10.7759/cureus.36196 PMC10104682

[11] Kattan AE AlShomer F Alhujayri AK Addar A Aljerian A. Current knowledge of burn injury first aid practices and applied traditional remedies: a nationwide survey. Burns Trauma. 2016;4:37. 27826592 10.1186/s41038-016-0063-7 PMC5094133

[12] Pencle F M.M. Zulfiqar H.. First degree burn. 2023. https://www.ncbi.nlm.nih.gov/books/NBK442021/. Accessed May 15, 2025. 28723050

[13] Jeschke MG van Baar ME Choudhry MA Chung KK Gibran NS Logsetty S. Burn injury. Nat Rev Dis Primers. 2020;6:11. 32054846 10.1038/s41572-020-0145-5 PMC7224101

[14] Greenhalgh DG. Management of burns. N Engl J Med. 2019;380:2349–59. 31189038 10.1056/NEJMra1807442

[15] Zakariya YF. Cronbach’s alpha in mathematics education research: its appropriateness, overuse, and alternatives in estimating scale reliability. Front Psychol. 2022;13:1074430. 36619096 10.3389/fpsyg.2022.1074430 PMC9813591

[16] Private Hospital Association Jordan. An overview of the Jordanian health sector. 2022. https://phajordan.org/EN-article-3809-#:~:text=The%20abundance%20of%20qualified%20medical%20and%20nursing%20cadres,nurses%20to%20provide%20health%20care%20services%20to%20patients. Accessed June 30, 2025.

[17] Dhanraj P. Management of burns. J Med Sci. 2017;3:102–10.

[18] Evers LH Bhavsar D Mailänder P. The biology of burn injury. Exp Dermatol. 2010;19:777–83. 20629737 10.1111/j.1600-0625.2010.01105.x

[19] Tolles J. Emergency department management of patients with thermal burns. Emerg Med Pract. 2018;20:1–24. 29369586

[20] Mortada H Malatani N Aljaaly H. Knowledge & awareness of burn first aid among health-care workers in Saudi Arabia: are health-care workers in need for an effective educational program? J Family Med Prim Care. 2020;9:4259–64. 33110842 10.4103/jfmpc.jfmpc_811_20 PMC7586629

[21] Mohammed NA Kadir SA. Assessment of nurses’ knowledge regarding initial management of burn victims in Kirkuk Hospitals. J Curr Med Res Opin. 2024;7:1226–31.

[22] Viana FO Eulálio KD Moura LKB Ribeiro IP Ramos CV. Primary health care professionals’ knowledge about initial care for burn victims. Rev Bras Enferm. 2020;73:e20180941. 32578731 10.1590/0034-7167-2018-0941

[23] Almutairi A. Burn injury characteristics and outcomes among pediatric and adult patients admitted to ministry of national guard health affairs (MNGHA) Hospitals in Saudi Arabia. Burns Open. 2023;7:146–52.

[24] Eftekhari H Sadeghi M Mobayen M. Epidemiology of chemical burns: an 11-year retrospective study of 126 patients at a referral burn centre in the north of Iran. Int Wound J. 2023;20:2788–94. 36931904 10.1111/iwj.14155 PMC10410324

[25] Wang Y Yu X Qian W. Epidemiologic investigation of chemical burns in Southwestern China from 2005 to 2016. J Burn Care Res. 2018;39:1006–16. 29939259 10.1093/jbcr/iry032

[26] Murari A Singh KN. Lund and Browder chart-modified versus original: a comparative study. Acute Crit Care. 2019;34:276–81. 31795625 10.4266/acc.2019.00647 PMC6895471

[27] Tran DP Arnold DH Thompson CM Richmond NJ Gondek S Kidd RS. Evaluating discrepancies in percent total body surface area burn assessments between prehospital providers and burn center physicians. J Burn Care Res. 2022;43:225–31. 34289051 10.1093/jbcr/irab131

[28] Minnesota Department of Health. Determining total body surface area. 2024. https://www.health.state.mn.us/communities/ep/surge/burn/tbsa.html. Accessed May 14, 2025.

[29] Guilabert P Usúa G Martín N Abarca L Barret JP Colomina MJ. Fluid resuscitation management in patients with burns: update. Br J Anaesth. 2016;117:284–96. 27543523 10.1093/bja/aew266

[30] World Health Organization. Basic emergency care: approach to the acutely Ill and injured. 2018. https://hlh.who.int/docs/librariesprovider4/clinical-care/who-icrc-basic-emergency-care.pdf. Accessed May 14, 2025.

[31] Greenhalgh DG. Burn resuscitation: the results of the ISBI/ABA survey. Burns. 2010;36:176–82. 20018451 10.1016/j.burns.2009.09.004

[32] Kalingamudali YT Jalini P Jayamaha A Jayaratne DL. Knowledge, attitudes, and practices on the usage of antiseptics prior to invasive medical procedures: evidence from Sri Lankan Healthcare Professionals. SAGE Open Med. 2023;11:20503121231199654. 37750100 10.1177/20503121231199654 PMC10518135

[33] Taddonio TE Thomson PD Smith DJ Prasad JK. A survey of wound monitoring and topical antimicrobial therapy practices in the treatment of burn injury. J Burn Care Rehabil. 1990;11:423–7. 2246312 10.1097/00004630-199009000-00009

[34] World Health Organization. Standards and Recommendations for Burns Care in Mass Casualty Incidents. 2024. https://iris.who.int/bitstream/handle/10665/379543/9789240100237-eng.pdf?sequence=1. Accessed May 14, 2025. 39591480

[35] Ousey K Rippon M Stephenson J. Barriers to wound debridement: results of an online survey. Wounds UK. 2016;12:36–41.

[36] American Burn Association (ABA). Guidelines for Burn Patient Referral. 2023. https://ameriburn.org/wp-content/uploads/2023/01/one-page-guidelines-for-burn-patient-referral-16.pdf. Accessed May 15, 2025.

[37] El-Helbawy RH Ghareeb FM. Inhalation injury as a prognostic factor for mortality in burn patients. Ann Burns Fire Disasters. 2011;24:82–8. 22262965 PMC3230152

[38] Pal N. Emergency escharotomy: overview, indications, contraindications. 2023. https://emedicine.medscape.com/article/80583-overview. Accessed May 15, 2025.

[39] Tasleem S Siddiqui AI Zuberi MAW. Mortality patterns and risk factors in burn patients: a cross-sectional study from Pakistan. Burns Open. 2023;8:13–8.

[40] Jeschke MG Pinto R Kraft R. Morbidity and survival probability in burn patients in modern burn care. Crit Care Med. 2015;43:808–15. 25559438 10.1097/CCM.0000000000000790 PMC4359665

[41] Rahimpour A Fox N Anderson J. Impact of total body surface area burn injuries on clinical outcomes and comorbidities in elderly patients aged over 65. Cureus. 2024;16:e76253. 39845211 10.7759/cureus.76253 PMC11753819

[42] Ghahghai M Ghorbani SS Hoseininejad S Sheikhi A Farhadi M Rahbar R. Factors responsible for mortality among burns patients in Islamic Republic of Iran. East Mediterr Health J. 2023;29:650–6. 37698220 10.26719/emhj.23.092

[43] Gete BC Mitiku TD Wudineh BA Endeshaw AS. Knowledge, attitude, and practice towards burn first aid and its associated factors among caregivers attending burn units in Addis Ababa, Ethiopia. A cross-sectional study. Ann Med Surg (Lond). 2022;81:104402. 36147091 10.1016/j.amsu.2022.104402 PMC9486655

[44] Hegde P Gibikote S Kumar A Thenmozhi M Jehangir S. Knowledge of prevention and first aid in burn injuries among health care workers and non-health care persons in India. Burns. 2024;50:1024–9. 38280840 10.1016/j.burns.2024.01.011

[45] Lam NN Li F Tuan CA Huong HTX. To evaluate first aid knowledge on burns management amongst high risk groups. Burns Open. 2017;1:29–32.

[46] Alhomaidan HT Rasheed Z Alsudais MM. Physicians based emergency medical services for the management of burn injuries in trauma centers of the center region of Saudi Arabia: evaluation of physicians’ knowledge and experience. Int J Burns Trauma. 2021;11:184–90. 34336383 PMC8310865

